# Association between total-Tau and brain atrophy one year after first-ever stroke

**DOI:** 10.1186/s12883-017-0890-6

**Published:** 2017-06-05

**Authors:** Hege Ihle-Hansen, Guri Hagberg, Brynjar Fure, Bente Thommessen, Morten W. Fagerland, Anne R. Øksengård, Knut Engedal, Per Selnes

**Affiliations:** 1 Department of Internal medicine, Vestre Viken Hospital Trust, Baerum Hospital, Norway, 3004 Drammen, Norway; 2 Norwegian Knowledge Centre for the Health Services, Oslo, Norway; 3 Department of Neurology, Akershus University Hospital, Lørenskog, Norway; 4 Unit of Biostatistics and Epidemiology, Oslo University Hospital, Oslo, Norway; 5 Norwegian Centre for Dementia Research, Oslo University Hospital, Oslo, Norway

**Keywords:** Stroke, Neurodegeneration, Cerebral spinal fluid

## Abstract

**Background:**

Although the most serious consequence of neuronal ischemia is acute neuronal death, mounting evidence suggests similarities between stroke and neurodegenerative disease. Brain atrophy visualized on structural MRI and pathological cerebrospinal fluid (CSF) concentrations of microtubule-associated protein tau (T-tau) and phosphorylated microtubule-associated protein tau indicate neurofibrillary degeneration. We aimed to explore the association between CSF T-tau and brain atrophy 1 year post-stroke.

**Methods:**

We included 210 patients with first-ever ischemic stroke or transitory ischemic attack without pre-existing cognitive impairment. After 12 months, subjects underwent MRI, and CSF biomarkers were assessed. Using SIENAX (part of FSL), ventricular CSF volume and total brain volume were estimated and normalized for subject head size. The association between T-tau as explanatory variable and ventricular and total brain volume as outcome variables were studied using linear regression.

**Results:**

One hundred eighty-two patients completed the follow-up. Forty-four had a lumbar puncture. Of these, 31 had their MRI with identical scan parameters. Mean age was 70.2 years (SD 11.7). Ventricular volume on MRI was significantly associated with age, but not with gender. In the multiple regression model, there was a significant association between T-tau and both ventricular (beta 0.44, 95% CI 376.3, 394.9, *p* = 0.021) and global brain volume (beta −0.50, 95% CI −565.9, −78.3, *p* = 0.011). There was no significant association between CSF T-tau 1 year post-stroke and baseline volumes.

**Conclusion:**

T-tau measured 1 year post-stroke is associated with measures of brain atrophy. The findings indicate that acute stroke may enhance or trigger tau-linked neurodegeneration with loss of neurons.

**Trial registration:**

Clinicaltrials.gov NCT00506818, July 23, 2007.

Inclusion from February 2007, randomization and intervention from May 2007 and trial registration in July 2007.

## Background

Although the most serious consequence of neuronal ischemia is acute neuronal death, mounting evidence suggests similarities between stroke and neurodegenerative diseases such as Alzheimer’s disease (AD) [1–4]. Stroke considerably increases the risk of dementia, up to 50% will develop dementia post-stroke and stroke is more common in patients with dementia [5–7]. Stroke, vascular dementia and AD have many shared risk factors, and there seems to be an interaction between ischemia, amyloid deposition and neuroinflammation, resulting in overlapping processes [8].

Established biomarkers of AD are cerebrospinal fluid (CSF) amyloid-β peptide (Aβ)-42 from amyloid plaques, CSF total microtubule-associated protein tau (T-tau) and phosphorylated microtubule-associated protein tau (P-tau). CSF Aβ-42 is the central biomarker of amyloid plaque formation, whereas T-tau and P-tau reflect neurofibrillary pathology. Whereas P-tau is considered more specific for AD, CSF T-tau is considered a more unspecific marker of axonal degeneration [9]. CSF T-tau is significantly increased the first days after ischemic stroke, peaking after 1–3 weeks with an apparent normalization after 3–5 months, and the magnitude of the increase correlates with infarct volume [10]. Atrophy (as measured by structural MRI) and T-tau are generally considered to indicate neurofibrillary degeneration, whereas decreased concentrations of CSF Aβ-42 are considered to represent amyloid deposition [11]. Hippocampal and cortical atrophy is well-described in both AD and pure cerebrovascular disease [12–14].

Most conditions predisposing for stroke, such as hypertension, diabetes, smoking and carotid stenosis, alter cerebral blood vessels and can cause inflammation, and the stroke itself further enhances this process [15]. In acute stroke the infarct core suffers acute cell death, whereas the penumbra is tissue at risk if the reperfusion remains insufficient. Cerebral hypoxia is associated with lower CSF levels of amyloid precursor protein (APP) metabolites [16]. Hypoxia is also shown to upregulate β-secretase activity associated with amyloid deposition and neuritic plaque formation [17]. Ischemia further affects the cytoskeleton by disrupting the normal function of microtubule associated proteins, notably tau phosphorylation, the normal regulating mechanism of microtubule associated protein tau [18–22]. Excitotoxicity is a common mechanism in many CNS diseases, referring to glutamate receptor overstimulation, secondarily disrupting intracellular calcium homeostasis. Excitotoxicity is central to the pathophysiologic mechanism of both ischemia and various neurodegenerative disorders [23]. Changes in the phosphorylation of tau would be only one of several effects of the calcium homeostasis disturbance [20].

Not only chronic ischemia, but also transient ischemic incidents with subsequent reperfusion are likely to lead to delayed cell damage. Experimental animal studies demonstrate immediate [24] and long-term disturbances in tau phosphorylation, causing axonal changes and neurofibrillary tangles (but not detectable amyloid plaques) in the ischemic areas [25]. Neurofibrillary tangles are first seen in the parahippocampal areas before appearing in the hippocampus proper [26]. Indeed, one recent study in young subjects found that first-ever stroke was associated with remote long-term hippocampal injury [27]. Biomarkers for post-stroke cognitive impairment overlap with biomarkers for neurodegeneration and ideally reflect disease-related pathological processes.

It has been difficult to determine the impact of stroke on cognitive decline, and the pathophysiological mechanisms involved in cognitive decline post-stroke remain incompletely understood. Because multiple lines of evidence indicate that acute and chronic ischemia may disrupt tau function and lead to neurodegeneration with ensuing cerebral atrophy, we hypothesized an association between CSF T-tau 1 year post-stroke and measures of brain atrophy. Thus, we aimed to explore the association between T-tau and brain atrophy, measured as ventricular volume and total brain volume, 1 year post-stroke.

## Methods

### Participants

We included all patients consecutively with first-ever ischemic stroke or transitory ischemic attack (TIA) without pre-existing cognitive decline in a randomized controlled trial (RCT) with change in cognition as the primary endpoint [28]. The study was registered in Clinicaltrials.gov (NCT00506818). The RCT was negative and the present study is analysed as a cohort. All patients were admitted to the stroke unit at Bærum Hospital, Vestre Viken Hospital Trust, and recruited between 2007 and 2008. Follow-up examinations were performed through 2009. Exclusion criteria were intracerebral haemorrhages, mild cognitive impairment or dementia diagnosed before the stroke onset, a history of cognitive decline as indicated by a score of ≥3,7 on the Informant Questionnaire on Cognitive Decline in the elderly (IQ-CODE) [29], previous stroke or TIA. Patients not fluent in Norwegian and patients with a remaining life expectancy of less than 1 year were not included. Details can be found in a previous published paper [28].

### Assessment

#### Baseline

Baseline examination included assessment of vascular risk factors (hypertension, hyperlipidemia, diabetes, body mass index, smoking and atrial fibrillation). Neurological impairment was assessed using the National Institutes of Health Stroke Scale (NIHSS) [30]. Activities of daily living were assessed by the Barthel ADL index [31], and global functioning was evaluated by the modified Rankin Scale [31], both at hospital admission and discharge. Cognitive function was measured between day 3 and 7 after admittance with the Mini Mental State Examination (MMSE) [32], Clock Drawing Test [33], Trail Making Test (TMT) A and B [34] and the immediate and delayed recall parts of the 10 word memory test (minimum score zero and maximum 40) [35]. Stroke classification was made according to The Trial of Org 10,172 in Acute Stroke Treatment (TOAST) [36] and the Oxfordshire Community Stroke Project classification (OCSP) [37] by a stroke physician. CT and/or MRI of the brain were performed in the acute phase, but not lumbar puncture.

### 12-month follow-up

At follow-up after 12 months, the patients underwent MRI of the brain. MRI scans were acquired on a Philips Intera system (Philips Medical Systems, Best, The Netherlands). At 1.5 T, an axial 2D spin echo sequence with the following sequence parameters was acquired: repetition time/echo time/inversion time (TI)/flip angle (FA) = 540 ms/14 ms/ms/90°, matrix =256 × 256, 22 slices, thickness = 5 mm, spaced at 6 mm. The MRI was performed according to the standards of the Radiological Department. There was a change of protocol during the inclusion period. Only subjects scanned with the protocol described below were included in the present study.

Ventricular CSF volume and total brain volume were estimated and normalized for subject head size with SIENAX [38, 39], part of FSL [40]. Firstly, SIENAX extracts brain and skull images from whole-head input data [41]. The brain-extracted image is further affine-registered to MNI152 space [42, 43], using the whole-head image to determine the registration scaling. This is primarily in order to obtain the volumetric scaling factor, to be used for head size normalization. Then, tissue-type segmentation with partial volume estimation is performed [44], calculating total volume of brain tissue. Separate estimates of tissue types are available; herein only ventricular CSF volume was used in addition to total brain volume.

Samples of CSF were collected by lumbar puncture through the L3-4 or L4-5 intervertebral space, in accordance with a standardized procedure, 12 months post-stroke. The lumbar puncture was performed at a standardized time of day (around noon), collected in polypropylene tubes and immediately transported to the local laboratory.

### Statistics

The associations between T-tau as explanatory variable and ventricular and total brain volume as outcome variables were estimated with linear regression analyses. As secondary analyses, we examined the relationships between CSF Aβ-42 and the volume variables. First, unadjusted analyses were performed. In the subsequent adjusted analyses, we included age and gender to the models, and, finally, Apolipoprotein E genotype (ApoEε4). We made plots for the prediction of the volume variables based on the linear regression models adjusted for age. The impact of individual observations was investigated by calculating the Cook’s D measure. A Cook’s D value above 1.0 may indicate an observation with great influence. In our models, the maximum value was 0.62, and we concluded that no observation had an undue influence on the results. The statistical analyses were performed with Stata 14 (StataCorp LP, College Station, TX).

## Results

**Fig. 1:**
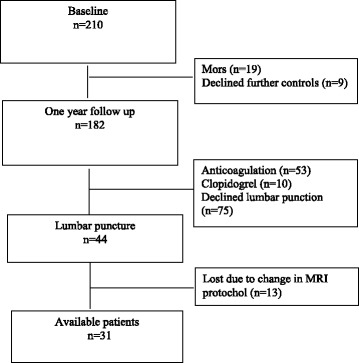
Flow diagram

In all, 210 patients were included, and 182 patients completed the 1-year follow-up. Of these, 44 underwent lumbar puncture at follow-up and 31 underwent MRI with identical scan parameters (Figure 1). Mean age of the 31 included patients was 70.2 years (SD 11.7). Baseline characteristics are listed in Table 1.

**Table 1:** Baseline characteristics of the 31 patients

Demographics	Baseline	Follow-up
Male, n	19 (61%)	
Mean age + −SD, years 70.2 (SD 11.7)		
< 9 years of education	5 (16%)	
Stroke subtype		
Cerebral infarction	29 (94%)	
TIA	2 (6%)	
Risk factors, n		
Hypertension	20 (65%)	
Hyperlipideamia	23 (74%)	
Diabetes	6 (19%)	
Current smoking	7 (23%)	
Atrial fibrillation	3 (10%)	
BMI	18 (58%)	
TOAST classification, n		
Large-vessel disease	2 (7%)	
Cardio-embolic disease	2 (6%)	
Small vessel disease	17 (55%)	
Stroke of undetermined aetiology	10 (32%)	
OCSP classification, n		
TACI	0	
PACI	13 (42%)	
LACI	15 (48%)	
POCI	3 (10%)	
Assessments		
NIHSS, day 1, mean (IQR)	3.06 (1.0-4.0)	0.58 (0.0-1.0)
BI, mean(IQR)	18.5 (19.5-20.0)	19.48 (20.0-20.0)
mRS, mean (IQR)	1.35 (0.0-3.0)	1.06 (1.0-1.0)
Total TAU, mean (IQR)		363 (227–382)
Apoɛ4	9 (29%)	
TMT A (seconds) (SD)	86.0 (±97.0)	61.6 (±48.4)
MMSE (points) (SD)	26.0 (±3.8)	26.7 (±3.9)
10 word test (words)	20.1 (±6.8)	24.5 (±6.1)
Ventricular volume (mm^3)(SD)	60,796 (±17,339)	60,334 (±25,118)
Global brain volume(mm^3) (SD)	1,404,660 (±63,160)	1,368,194 (±142,856)

TIA = Transient Ischemic Attack; Hyperlipidemia = total cholesterol >5 mmol/l or LDL-cholesterol >3 mmol/l; LDL = Low Density Lipoprotein; BMI = body mass index; TOAST = The Trial of Org 10,172 in Acute Stroke Treatment classification; OCSP = Oxfordshire Community Stroke Project classification; TACI = Total Anterior Circulation Infarction; PACI = Partial Anterior Circulation Infarction; LACI = Lacunar Circulation Infarction; POCI = Posterior Circulation Infarction; NIHSS = National Institute of Health Stroke Scale; IQR = Interquartile range; BI = Barthel Activities of Daily Living Index; mRs = modified Rankin scale; Apoɛ4 = apo lipoprotein E allel 4; TMT A = trail making test A; SD = standard deviation); MMSE = mini metal test examination

**Fig. 2:**
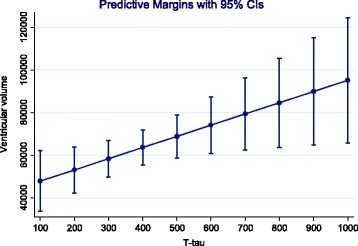
The relationship between T-tau and ventricular volume. Predictive margins with 95%

**Fig. 3:**
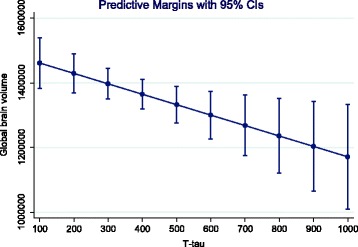
The relationship between T-tau and global volume. Predictive margins with 95%

MRI volume variables on ventricular volume 1 year post-stroke were significantly associated with age (beta 0.39, 95% CI 87.1,1718, *p* = 0.031), but not with gender (beta −0.61, 95% CI −23,871, 17,294, *p* = 0.75). MRI volume variables on global brain volume was not significantly associated with age (beta −0.31, 95% CI −8524, 665.1, *p* = 0,091), or gender (beta −0.04, 95% CI −123,912, 101,187, *p* = 0.84). In the multiple regression model, there was a significant correlation between T-tau and both ventricular volume (beta 0.44, 95% CI 376.3, 394.9, *p* = 0.021) and global brain volume (beta −0.50, 95% CI −565.9, −78.3, *p* = 0.011) 1 year post-stroke. Predictive margins of T-tau on volume variables are shown in Figs. 2 and 3. The analyses that included ApoEε4 in the regression models did not change the results (data not shown).

Twenty eight (63%) of the patients underwent MRI during the acute phase. There was no significant association with MRI volume at baseline and T-tau 1 year later. In addition, there were no correlations between CSF T-tau (controlling for age) and measures of cognition (all at follow-up).

For the secondary analysis on CSF Aβ-42 and ventricular or global volume, no significant associations were found ((beta −0.17, 95% CI −47.2, 17.3, *p* = 0.35) and (beta −0.01, 95% CI −190.9, 178.1, *p* = 0.95)).

## Discussion

One year after first-ever stroke, we found a significant association between T-tau and measures of brain atrophy like ventricular and total brain volume. There was no significant association between CSF T-tau (1 year post-stroke) and baseline ventricular and brain volume. The present results suggest that acute stroke may trigger or enhance neurodegeneration with accompanying increased T-tau.

The microvasculature in neurodegeneration and cerebrovascular disease present with several distinct pathological changes. Endothelial cells and surrounding pericytes degenerate and become atrophic, the capillary basement membrane thicken and protrude into the capillary lumen and result in gradually decreased cerebral blood flow [4, 45]. The present results may be construed to represent the stroke triggering mechanisms leading to atrophy. Alternatively, there may be an interaction between the acute event and the preexisting vulnerability, or even a combination. The reperfusion injury may be enhanced by microvascular changes, leading to a further decrease in oxygen availability.

Twenty subjects were scanned with the same protocol at baseline and follow-up. Numerically, the MRI volume variables were smaller at follow-up, but there was no significant change from baseline to follow-up. Not controlling for age, there were highly significant correlations between volume change and CSF T-tau at follow-up. When controlling for age, however, there was a significant correlation between increase in ventricular volume and CSF T-tau, but no significant correlation between whole brain volumes and CSF T-tau. The limited number of subjects scanned at both baseline and follow-up preclude conclusions based on these observations, but putatively first-ever stroke may initiate or enhance an ongoing neurodegenerative process.

Biomarkers of neurodegeneration (e.g. increased CSF T-tau and cerebral atrophy) are expected to appear before functional deficits [11], and the baseline cognitive measurements tested here are crude and with a significant expected variability. The baseline cognitive testing was performed during acute illness and hospitalization, likely to affect these results. The cognitive variables showed a general improvement from baseline to follow-up. Accordingly, we did not examine the relationship between change in cognitive variables from baseline to 1 year follow-up and CSF T-tau or atrophy.

Our study has several limitations. Small sample size, younger population and milder strokes compared with the general stroke patients are some. In addition, it is difficult to motivate patients in a stable phase after the stroke to undergo lumbar puncture. Patients with atrial fibrillation on oral anticoagulation were excluded due to safety concerns. Therefore, patient with possible more severe stroke with cardio embolic etiology and higher risk for cognitive impairment are not included in the analyses. The MRI protocol was changed during the study, resulting in further exclusions.

## Conclusions

In order to start interventions that may improve cognition or prevent progression at an early stage, we need more knowledge regarding mechanisms involved in post-stroke brain changes. The results from this study raise the hypothesis that tau-linked neurodegeneration may be of crucial pathophysiological importance after stroke. This study also implicates that MRI with measurements of atrophy may be of importance in post-stroke follow-ups, regarding sustained neurodegeneration.

## Abbreviations

Aβ-42: Amyloid-β peptide-42
AD: Alzheimer’s disease
ApoE: Apolipoprotein E genotype
APP: Amyloid precursor protein
CSF: Cerebrospinal fluid
IQ-CODE: Informant Questionnaire on Cognitive Decline in the elderly
MMSE: Mini Mental State Examination
NIHSS: National Institutes of Health Stroke Scale
OCSP: Oxfordshire Community Stroke Project classification
P-tau: Phosphorylated microtubule-associated protein tau
RCT: Randomized controlled trial
TIA: Transitory ischemic attack
TOAST: The Trial of Org 10,172 in Acute Stroke Treatment
TMT A: Trail Making Test A
TMT B: Trail Making Test B
T-tau: Microtubule-associated protein tau
